# Characterization of *bla*_NDM–5_-carrying plasmids in two clinical *Salmonella* isolates from Jiaxing city, China

**DOI:** 10.3389/fmicb.2025.1620907

**Published:** 2025-06-26

**Authors:** Ping Li, Yongjuan Yuan, Yong Yan, Miaomiao Jia, Lei Gao, Xuejuan Liu, Yangming Sun, Guoying Zhu, Zhongwen Chen

**Affiliations:** ^1^Jiaxing Center for Disease Control and Prevention, Jiaxing, China; ^2^Jiashan County Center for Disease Control and Prevention, Jiaxing, China

**Keywords:** non-typhoidal *Salmonella*, carbapenem-resistant, IncHI2/IncHI2A, IS*26* unit, *bla*_NDM–5_

## Abstract

**Introduction:**

*Salmonella* is an important cause of foodborne diarrheal diseases worldwide. The emergence of *bla*_NDM_-positive carbapenem-resistant *Salmonella enterica* isolates in recent years poses a huge public health challenge.

**Methods:**

In this study, two clinical *S. enterica* isolates carrying *bla*_NDM–5_: a serotype 4,[5],12:i:- strain (2023JX045) and a serovar Stanley strain (2024–406) were analyzed using antimicrobial susceptibility testing and whole genome sequencing.

**Results:**

Both isolates were multidrug resistant, with insusceptibility to ampicillin, ampicillin/sulbactam, amoxicillin/clavulanic acid, cefuroxime, ceftiofur, cefazolin, cefoxitin, cefotaxime, ceftazidime, cefepime, ceftazidime/avibactam, meropenem, imipenem, ertapenem, tetracycline, gentamicin, trimethoprim/sulfamethoxazole, florfenicol, chloramphenicol, ciprofloxacin, colistin, and polymixin B. The *bla*_NDM–5_-carrying plasmids in 2023JX045 and 2024–406 were named p23045-NDM5 and p2024406-NDM5, respectively, with both belonging to the incompatibility (Inc)HI2/IncHI2A group and sequence type ST3. In 2023JX045, *bla*_NDM–5_ was flanked by two same-oriented copies of IS*26* elements (IS*26-dsbC-trpF*-*ble*_MBL_-*bla*_NDM–5_-IS*5*-ΔIS*3000*-ΔIS*Kox3-umuC-umuD*-IS*26*). In 2024–406, p2024406-NDM5 was found to carry two copies of *bla*_NDM–5_, possibly resulting from duplication of IS*26*-*dsbC-trpF*-*ble*_MBL_-*bla*_NDM–5_-IS5-ΔISAba125-ΔIS3000-ΔISKox3-umuC-umuD-IS26 and interrupted by mobile element IS*1* at the upstream region of ΔIS3000.

**Discussion:**

This is the first report to describe the presence of two *bla*_NDM–5_ copies on an IncHI2/IncHI2A plasmid carried by serovar Stanley, as well as the dissemination of *bla*_NDM–5_ in Salmonella in Jiaxing City, China. IS26-flanked composite transposons appeared to play an important role in the formation of this region. The dissemination of blaNDM in Salmonella isolates and the complexity of the *bla*_NDM–5_ region highlight the urgent need to monitor carbapenem-resistant *S. enterica*.

## 1 Introduction

Non-typhoidal *Salmonella* (NTS) is one of the most prevalent foodborne pathogens, consistently causing gastrointestinal infections. More than 2,600 serovars of NTS have been identified, among which *S*. Typhimurium is one of the most common (Lamichhane et al., 2024). Previous studies have indicated that *Salmonella enterica* may act as a reservoir for carbapenemase genes, contributing to the transmission of carbapenem resistance via the food chain (Day et al., 2015). Whole-genome sequencing (WGS) data of global carbapenem-resistant *S. enterica* (CRSE) isolates revealed *S*. Typhimurium (21.8%) to be the most prevalent serotype of CRSE worldwide (Wu et al., 2023). Additionally, Typhimurium (25.8%) and Senftenberg (19.4%) were the most prevalent serovars of global CRSE isolates harboring the New Delhi metallo-β-lactamase (*bla*_NDM_) gene (Zhao et al., 2025). In recent years, the *bla*_NDM_ gene has been identified in *S*. Stanley isolated from clinical and environmental samples (Deng et al., 2024, Huang et al., 2013).

The worldwide spread of multidrug-resistant (MDR) *Enterobacteriaceae* strains, particularly carbapenem-resistant *Enterobacteriaceae* (CRE), has become an increasing public health threat (Nordmann et al., 2012). Mobile resistance elements carrying *bla*_NDM–1_ have contributed to the dramatic increase in the prevalence of CRE in clinical settings (Huang et al., 2016). Twenty-nine NDM protein variants have been identified since 2009 (Mojica et al., 2022). The *bla*NDM-5 gene was first identified in a clinical *Escherichia coli* strain (EC045) from India in 2011, and was later commonly identified among strains of *E. coli* (Yang et al., 2014, Sassi et al., 2014), *Klebsiella pneumoniae* (Bathoorn et al., 2015), and *Morganella morganii* (Guo et al., 2019). In China, a report on *bla*_NDM–1_ in Acinetobacter baumannii isolates appeared in early 2011 (Chen et al., 2011). Among *Salmonella* spp., *bla*_NDM–1_ was first identified in a Senftenberg isolate in 2011, and was located on an incompatibility (Inc)L/M group plasmid (Savard et al., 2011, Rasheed et al., 2013). A 2012 report described the identification of a *bla*_NDM–1_-bearing strain of *Salmonella* Stanley isolated from the feces of an 11-month-old girl (Huang et al., 2013). The first *bla*_NDM–5_-positive IncFII plasmid, isolated from an *S*. Typhimurium sequence type (ST) 34 isolate in China, was reported in 2015 (Li et al., 2017). Compared with *bla*_NDM–1_, *bla*_NDM–5_ has two amino acid substitutions (Val88Leu and Met154Leu) and confers a high level of resistance to carbapenems and broad-spectrum cephalosporins (Hornsey et al., 2011).

Here, we aimed to better understand the antimicrobial resistance determinants and transmission risk of *bla*_NDM_-positive *Salmonella* in Jiaxing City, China by conducting a comprehensive investigation of two *bla*_NDM–5_-carrying carbapenem-resistant *Salmonella* isolates that were recovered from clinical samples. To the best of our knowledge, this is the first report of *Salmonella* isolates carrying *bla*_NDM_ in this part of China. Notably, it is also the first report of an IncHI2/IncHI2A plasmid co-carrying two copies of *bla*_NDM–5_ in *S*. Stanley. Jiaxing is located in the Yangtze River Delta region, with well-developed water and land transportation and a continuously growing population. It is an important economic and population aggregation area in Zhejiang Province. These findings further complicate the challenges of establishing effective treatment modalities and management strategies.

## 2 Materials and methods

### 2.1 Bacterial collection and characterization

Fecal samples from patients with acute clinical diarrhea were collected to isolate *Salmonella* spp. Within 4 h of collection, undiluted samples were streaked onto Columbia Blood Agar plates (CHROMagar, Shanghai, China) and cultured overnight at 37°C. Suspected *Salmonella* spp. colonies were analyzed using matrix-assisted laser desorption/ionization–time of flight mass spectrometry. Serotyping was conducted using the slide agglutination method to detect somatic (O) antigen and flagellar (H) antigens (phase 1 and 2) following the White–Kaufmann–Le Minor Scheme. *Salmonella* Serotyping by Whole Genome Sequencing was confirmed using the Sequence query tool implemented in SeqSero2/SeqSero2S.^1^

### 2.2 Antimicrobial susceptibility testing

AST of the following antimicrobial agents was performed to determine the minimum inhibitory concentration (MIC) of each using the microdilution method: ampicillin, ampicillin/ sulbactam, amoxicillin/clavulanic acid, cefuroxime, ceftiofur, cefazolin, cefoxitin, cefotaxime, ceftazidime, cefepime, cefotaxime/ clavulanate, ceftazidime/clavulanic acid, ceftazidime/avibactam, meropenem, imipenem, ertapenem, tetracycline, gentamicin, amikacin, trimethoprim/sulfamethoxazole, florfenicol, chloramphenicol, ciprofloxacin, nalidixic acid, colistin, polymixin, and azithromycin. The resistance breakpoints of ampicillin, ceftiofur, imipenem, meropenem, ertapenem, azithromycin, tetracycline, ciprofloxacin, trimethoprim/sulfamethoxazole, and chloramphenicol were determined in accordance with the principles outlined in relevant documents from the Clinical and Laboratory Standards Institute (CLSI) (M100-S32, M45-A3). Amoxicillin/clavulanic acid, ampicillin/sulbactam, cefazolin, cefepime, cefotaxime, cefoxitin, ceftazidime, cefuroxime, ceftazidime/avibactam, gentamicin, and amikacin were determined in accordance with the European Committee on Antimicrobial Susceptibility Testing (EUCAST). Colistin, Polymixin B, and florfenicol were interpreted in accordance with the “National Food Contamination and Hazardous Factor Risk Monitoring Work Manual 2024” (China National Center for Food Safety Risk Assessment, 2024). *Escherichia coli* ATCC 25922, *Enterococcus faecalis* ATCC29212, *Pseudomonas aeruginosa* ATCC27853, and *Staphylococcus aureus* ATCC29213 was used as a quality control strains for AST.

### 2.3 Genomic DNA extraction and WGS

Total genomic DNA was extracted from overnight (16–18 h) cultures of strains 2023JX045 and 2024-406 using the QIAamp DNA Mini Kit (Qiagen, Hilden, Germany) following the manufacturer’s instructions. WGS was performed on the two strains using both the long-read Nanopore MinION (Nanopore, Oxford, United Kingdom) and the short-read NextSeq 550 (Illumina, San Diego, CA, United States) platforms. The derived short reads and long reads were assembled using SPAde v.3.6 software.

### 2.4 Bioinformatic analysis

The ST of *Salmonella* isolates were determined using multilocus sequence typing software.^2^ Antimicrobial-resistant genes and plasmid profiles were analyzed used ResFinder^3^ and PlasmidFinder.^4^ Annotation of mobile elements was carried out using online databases, such as ISfinder.^5^ Plasmid sequence alignment was performed using BRIG v0.95 (Alikhan et al., 2011) and Easyfig v2.2.5.^6^

To investigate the epidemic characteristics of *bla*_NDM_-carrying plasmids in *Salmonella*, we obtained 31 *bla*_NDM_-positive plasmids from the GenBank core nucleotide database (last accessed on 6th January, 2025. Plasmids from *Salmonella* isolates (taxid: 590) were selected). For the input sequences, multiple sequence alignment (MSA) was performed with the Multiple Alignment Using Fast Fourier Transform in auto mode. The resulting MSA was then entered into ModelTest using default parameters to estimate the best model for constructing the evolutionary tree. Subsequently, the MSA and the selected model were used as input for RAxML-NG with the arguments –all –seed 12345 –bs-trees 1000) to generate the final evolutionary tree. Visualization and annotation of the phylogenetic tree were performed using iTOL v7.^7^

### 2.5 Nucleotide sequence accession number

The sequences of plasmids p23045-NDM5 and p2024406-NDM5 were submitted to the GenBank database and assigned accession numbers OR497833 and PQ844496, respectively.

## 3 Results

### 3.1 Characterization of two carbapenem-resistant Salmonella strains

*S. enterica* strains 2023JX045 and 2024-406 were isolated from clinical diarrhea samples collected from a 20-month-old boy and 57-year-old woman, respectively. The main symptoms of case 1 were a fever of 39.3°C and watery diarrhea 10 times per day. No history of suspected food exposure was found. Case 2 did not have a fever. The digestive system symptoms were abdominal pain and watery diarrhea five times a day. It is suspected that it might be related to the consumption of bulk fruits and their products. The patients and his/her family had not traveled to any country in recent 7 days, and no family members were affected. Strain 2023JX045, identified as 4,[5],12:i:-, a ST34 monophasic variant of *S. enterica* serovar Typhimurium, was found to carry the following antimicrobial resistance genes: *aph(4)-Ia*, *aph(3’)-Ia*, *aac(3)-IV*, *aadA2b*, *bla*_NDM–5_, *bla*_OXA–10_, *bla*_TEM–1B_, *lnu(F)*, *qnrS1*, and *sul3*. Strain 2024-406 was identified as belonging to ST29 *S. enterica* serovar Stanley (4,12:d:2). The antimicrobial resistance genes identified in this isolate included the following: *aph(4)-Ia*, *aph(6)-Id*, *aph(3’)-Ia*, *aph(3”)-Ib*, *aadA1*, *aadA1*, *aadA2b*, *aac(3)-IV*, *bla*_TEM–1B_, *bla*_NDM–5_, *bla*_NDM–5_, *bla*_OXA–10_, *cmlA1*, *cmlA1*, *floR*, *qnrS1*, *ARR-2*, *sul3*, *tet(A)*, *tet(A)*, and *dfrA14*. Furthermore, 2024-406 was shown to possess a single point mutation in the quinolone resistance-determining region of *parC* (T57S). While a sole plasmid carrying IncHI2/IncHI2A replicons was identified in 2024-406, 2023JX045 was found to carry multiple plasmid replicons, namely Col (pHAD28), IncHI2/IncHI2A, IncI2 (Delta), IncQ1, and p0111 (Table 1).

**TABLE 1:** Information about the NDM-5-harboring *Salmonella* strains 2023JX045 and 2024-406 identified in this study and its plasmids.

Strain	Sampling date	patient	Serotype	Resistant profiles^a^	Resistance genes	Plasmid	
						Replicon type	Size of plasmid (bp) in this study
2023JX045	2023/04/23	Male (1 year old and 8 month)	Monophasic *Salmonella* Typhimurium (4,[5],12:i:-)	AMP, AMS, AMC, CXM, CEF, CFZ, CFX, CTX, CAZ, FEP, CZA, MEM, IPM, ETP, TET, GEN, SXT, FFC, CHL, CIP, CT, PB	*aph(4)-Ia*, *aph(6)-Id*, *aph(3’)-Ia*, *aph(3”)-Ib*, *aadA22*, *aadA1*, *aadA1*, *aadA2b*, *aac(3)-IV*, *aph(6)-Id*, *aph(3”)-Ib*, *aph(6)-Id*, *aph(3”)-Ib*, *bla*_TEM–1B_, ***bla*_NDM–5_**, *bla*_OXA–10_, *bla*_TEM–1B_, *bla*_TEM–1B_, *lnu(F)*, *cmlA1*, *cmlA1*, *floR*, *floR* *qnrS1*, *qnrS1*	IncHI2/IncHI2A (p23045-NDM5) Col(pHAD28) IncI2(Delta) IncQ1 p0111	p23045-NDM5: 266,011 bp
2024-406	2024/09/03	Female (57 years old)	*Salmonella* Stanly (4,12:d:2)	AMP, AMS, AMC, CXM, CEF, CFZ, CFX, CTX, CAZ, FEP, CZA, MEM, IPM, ETP, TET, GEN, SXT, FFC, CHL, CIP, CT, PB	*aph(4)-Ia*, *aph(6)-Id*, *aph(3’)-Ia*, *aph(3”)-Ib*, *aadA1*, *aadA1*, *aadA2b*, *aac(3)-IV*, *bla*_TEM–1B_, ***bla*_NDM–5_**, ***bla*_NDM–5_**, *bla*_OXA–10_, *cmlA1*, *cmlA1*, *floR*, *qnrS1*, *ARR-2*, *sul3*, *tet(A)*, *tet(A)*, *dfrA14*	IncHI2/IncHI2A (p2024406-NDM5)	277,574 bp

AMP, Ampicillin; AMS, Ampicillin/Sulbactam; AMC, Amoxicillin/Clavulanic Acid; CXM, Cefuroxime; CEF, Ceftiofur; CFZ, Cefazolin; CFX, Cefoxitin; CTX, Cefotaxime; CAZ, Ceftazidime; FEP, Cefepime; CZA, Ceftazidime/avibactam; MEM, Meropenem; IPM, Imipenem; ETP, Ertapenem; TET, Tetracycline; GEN, Gentamicin; SXT, Trimethoprim/Sulfamethoxazole; FFC, Florfenicol; CHL, Chloramphenicol; CIP, Ciprofloxacin; CT, Colistin; and PB, Polymixin B.

The AST analysis showed that these two isolates were MDR to β-lactams, including penicillins (ampicillin, ampicillin/sulbactam, and amoxicillin/clavulanic acid) and cephalosporins (cefuroxime, ceftiofur, cefazolin, cefoxitin, cefotaxime, ceftazidime, cefepime, and ceftazidime/avibactam), carbapenems (meropenem, imipenem, and ertapenem), tetracycline (tetracycline), aminoglycosides (gentamicin), sulfonamides (trimethoprim/sulfamethoxazole), amphenicols (florfenicol and chloramphenicol), fluoroquinolones (ciprofloxacin), and polymyxins (colistin and polymixin B), but remained susceptible to amikacin and azithromycin (Table 2).

**TABLE 2:** AST of the 2023JX045 and 2024-406 isolates using a panel of 27 antimicrobial agents.

Antibiotic type		Antimicrobial Agent	MIC (mg/L)/(R/I/S)	MIC (mg/L)/(R/I/S)
			2024-406	2023JX045
β-lactams	Penicillins	Ampicillin	> 64(R)	>64(R)
		Ampicillin/Sulbactam	> 64(R)	>64(R)
		Amoxicillin/Clavulanic Acid	64(R)	64(R)
	Cephalosporins	Cefuroxime	> 64(R)	>32(R)
		Ceftiofur	> 16(R)	>16(R)
		Cefazolin	> 32(R)	>32(R)
		Cefoxitin	> 64(R)	>64(R)
		Cefotaxime	> 8(R)	>16(R)
		Ceftazidime	> 32(R)	>32(R)
		Cefepime	> 32(R)	>32(R)
		Cefotaxime/Clavulanate	> 4(−)	>8(−)
		Ceftazidime/Clavulanic Acid	> 16(−)	>16(−)
		Ceftazidime/avibactam	> 16(R)	>8(R)
Carbapenems		Meropenem	> 4(R)	4(R)
		Imipenem	8(R)	2(I)
		Ertapenem	> 8(R)	8(R)
Tetracycline		Tetracycline	> 32(R)	>32(R)
Aminoglycosides		Gentamicin	32(R)	16(R)
		Amikacin	< = 4(S)	< = 4(S)
Sulfonamides		Trimethoprim/Sulfamethoxazole	> 8(R)	>8(R)
Amphenicols		Florfenicol	> 32(R)	>32(R)
		Chloramphenicol	> 64(R)	>64(R)
Fluoro-quinolones		Ciprofloxacin	0.5(I)	0.5(I)
		Nalidixic	8(-)	8(-)
Polymyxin		Colistin	< = 0.25(I)	0.5(I)
		Polymixin B	0.5(I)	0.25(I)
Macrolides		Azithromycin	4(S)	4(S)

R, Resistant; I, Intermediate; S, Susceptiable; −, Not applicable.

### 3.2 Plasmid characterization

The carbapenemase-encoding gene *bla*_NDM–5_ was found on a 266,011-bp plasmid (p23045-NDM5) with 47.1% GC content in strain 2023JX045. Strain 2024-406 was found to carry a 277,574-bp plasmid (p2024406-NDM5) with 47.3% GC content. Both p23045-NDM5 and p2024406-NDM5 were classified as IncHI2/IncHI2A and ST3 plasmids. Exhibiting 99% coverage and 100% identity with p23045-NDM5, p2024406-NDM5 is distinguished by numerous rearrangements and inversions within accessory regions.

The resistance genes in p23045-NDM5 were found to be arranged in three regions. The first region contains △*tet(A)*, *qnrS1*, *aadA22*, *lnu(F)*, *aph(3”)-Ib*, *aph(6)-Id*, *aph(3’)-Ia*, *aph(4)-Ia*, *aac(3)-Iva*, *sul3*, *aadA1*, *cmlA1*, and *aadA2b*, with the major antimicrobial resistance genes arranged within two class 1 integrons (IntI1). The *bla*_NDM–5_-region carries resistance genes including *floR*, *tet(A)*, *dfrA14*, *aadA1*, *bla*_OXA–10_, *cmlA*, *ARR-2*, *ble*_MBL_, and *bla*_NDM–5_. This plasmid also carries *bla*_TEM–1_ within a truncated Tn*2*.

In p2024406-NDM5, we found the resistance genes *aph(4)-Ia*, *aph(6)-Id*, *aph(3’)-Ia*, *aph(3”)-Ib*, *aadA1*, *aadA1*, *aadA2b*, *aac(3)-IV*, *bla*_TEM–1B_, *bla*_OXA–10_, *cmlA1*, *cmlA1*, *floR*, *qnrS1*, *ARR-2*, *sul3*, *tet(A)*, *tet(A)*, *dfrA14*, along with two copies of *bla*_NDM–5_ are clustered in a complicated accessory region. The *bla*_OXA–10_-region containing *tet(A)*, *qnrS1*, △ *bla*_TEM–1B_, *tet(A)*, *floR*, and a class 1 integron carrying *ARR-2*, *cmlA1*, *bla*_OXA–10_, *aadA1*, and *dfrA14* resistant genes.

**FIGURE 1:**
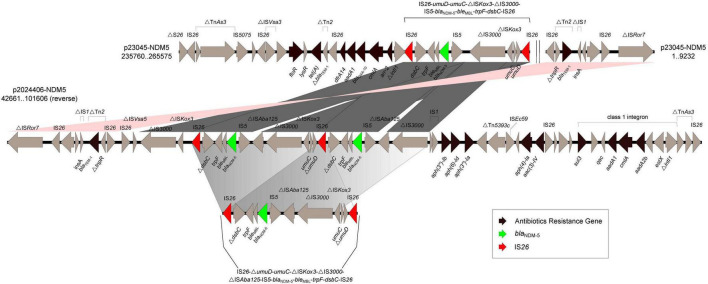
Schematic representation and comparison of the genetic environments of the *bla*_NDM_-flanking region in p23045-NDM5 and p2024406-NDM5 recovered from this study. Arrows indicate the direction of transcription of each gene. Numbers in brackets indicate nucleotide positions within corresponding plasmid sequences.

Compared with the genetic background of the typical *bla*_NDM5–_IncX3 plasmid pNDM_MGR194 (IS*3000*-△ IS*Aba125*-IS*5*-*bla*_NDM–5_-*ble*-*trpF*-*dsbC*-IS*26*), the *bla*_NDM–5_ gene on plasmid p23045-NDM5 was found to be flanked by two same-oriented copies of IS*26* elements (IS*26*-*umuD*-*umuC*-△ IS*Kox3*-△ IS*3000*-IS*5*-*bla*_NDM–5_-*ble*_MBL_-*trpF*-*dsbC*-IS*26*). Note the absence of the △ IS*Aba125* feature in this region. We identified more complex genetic arrangements in p2024406-NDM5, formed by duplication of the IS*26*-*umuD*-*umuC*-△ IS*Kox3*-△ IS*3000*-△ IS*Aba125*-IS*5*-*bla*_NDM–5_-*ble*_MBL_-*trpF*-*dsbC*-IS*26* unit. Additionally, mobile element IS*1* was found upstream of △ IS*3000*, which may have led to the loss of IS*26*-*umuD*-*umuC*-△ IS*Kox3* (Figure 1). A direct repeat was not detected immediately upstream or downstream of those two IS*26*-flanked regions.

### 3.3 Comparative study with other NDM-positive plasmids in *S. enterica* strains already identified

A total of 31 *S. enterica* strains with positivity for *bla*_NDM_ plasmids were obtained from the GenBank core nucleotide database (Table 3). The serovars of these strains, mostly isolated from *Homo sapiens*, were Senftenberg (*n* = 2), Bareilly (*n* = 1), London (*n* = 2), Lomita (*n* = 1), Enteritidis (*n* = 1), Mbandaka (*n* = 2), Rissen (*n* = 1), 1,4,[5],12:i:- (*n* = 11), Stanley (*n* = 2), Corvallis (*n* = 2), Kottbus (*n* = 1), 1,4,[5],12:i:2 (*n* = 2), Typhimurium (*n* = 1), and Indiana (*n* = 1). The most prevalent *bla*_NDM_ gene was *bla*_NDM–1_, followed by *bla*_NDM–5_, which was the major *bla*_NDM_ gene in 1,4,[5],12:i:- isolates. Two *S*. Stanley strains carried a *bla*_NDM_ gene, with one isolate harboring *bla*_NDM–1_ and the other *bla*_NDM–5_. Despite the presence of *bla*_NDM_, other β-lactamase genes (e.g., *bla*_CMY_, *bla*_OXA_, and *bla*_TEM_) were also detected in some of these strains.

**TABLE 3:** 31 *S. enterica* strains with positivity for *bla*_NDM_ plasmids obtained from the GenBank core nucleotide database.

Plasmid_name	serovar	Plasmid_length (bp)	Geo_loc_name	Collection date	Inc type	*bla*_NDM_	Other resistant genes	Accession No.
pNDM-SAL	Senftenberg	1,46,129	India	/	IncC	*bla*_NDM–1_	*bla*_CMY–4_, *aac(6’)-Ib3*, *sul1*	KP742988
pFC745	Bareilly	2,42,007	India: Vellore	2017/7	IncC	*bla*_NDM–7_	*bla*_CMY–6_, *bla*_TEM–1A_, *rmtC*, *aadA2*, *armA*, *aac(6’)-Ib3*, *msr(E)*, *mph(E)*, *sul1*, *sul1*, *dfrA12*	CP063685
pSAL-19-0623_NDM	London	2,76,695	Singapore	2019	IncC/IncFIB(K)	*bla*_NDM–1_	*bla*_OXA–4_, *aadA16*, *aph(6)-Id*, *aph(3”)-Ib*, *aph(3’)-VIa*, *aadA2b*, *aac(6’)-Ib-cr*, *ant(2”)-Ia*, *mph(A)*, *qnrA1*, *qnrB6*, *ARR-3*, *sul1*, *sul1*, *sul1*, *sul1*, *sul1*, *dfrA27*	MN604267
pSL131_IncA/C-IncX3	Lomita	2,16,895	China	/	IncC/IncX3	*bla*_NDM–1_	*bla*_CMY–2_, *aph(6)-Id*, *aph(3’)-Ia*, *aph(3”)-Ib*, *aadA2*, *mph(A)*, *floR*, *sul1*, *sul2*, *tet(A)*, *dfrA12*	MH105050
pIncCSEn	Enteritidis	1,68,240	Uruguay	/	IncC	*bla*_NDM–1_	*aph(3’)-Ia*, *aadA2*, *mph(A)*, *cmlA1*, *catA1*, *qnrA1*, *sul1*, *sul1*, *sul1*, *sul2*, *dfrA12*	PQ202990
pSM28_NDM_1	Mbandaka	2,10,622	China	2021/12/28	IncC	*bla*_NDM–1_/ *bla*_NDM–1_/*bla*_NDM–1_	*bla*_SFO–1_, *bla*_TEM–1B_, *aac(3)-IId*, *msr(E)*, *mph(A)*, *mph(E)*, *sul1*, *dfrA12*	CP138308
pSM30_NDM_1	Mbandaka	2,38,640	China	2021/12/30	IncC	7 copies of *bla*_NDM–1_	*bla*_SFO–1_, *bla*_TEM–1B_, *aac(3)-IId*, *msr(E)*, *mph(A)*, *mph(E)*, *sul1*, *dfrA12*	CP138306
pA132-1-NDM	London	1,14,617	China: Huzhou	2023/5/1	IncFIB(K)	*bla*_NDM–5_	*bla*_TEM–1B_, *aac(3)-IId*, *aadA16*, *aph(6)-Id*, *aph(3”)-Ib*, *aadA2*, *aac(6’)-Ib-cr*, *mph(A)*, *floR*, *qnrB6*, *ARR-3*, *sul1*, *sul1*, *sul1*, *sul2*, *tet(A)*, *dfrA12*, *dfrA27*	CP141257
pNDM13-SR33	Rissen	88,258	China: Xiamen, Fujian	2021/9/14	IncI1-I(Alpha)	*bla*_NDM–13_	/	CP092912
81741 plasmid unnamed2	1,4,[5],12:i:-	84,565	/	/	IncFII	*bla*_NDM–5_	*bla*_TEM–1B_, *mph(A)*	CP019444
pNDM-IncFII	1,4,[5],12:i:-	77,789	China: Guangzhou	2021/11/27	IncFII	*bla*_NDM–5_	*bla*_TEM–1B_, *mph(A)*	CP110199
sg1722-2 plasmid unnamed1	1,4,[5],12:i:-	84,884	China: Zhejiang	2021/5/15	IncFII(pCoo)	*bla*_NDM–1_	*qnrS1*	CP081190
pT2-4-4-ndm	1,4,[5],12:i:-	1,38,709	China	/	IncFII(pCoo)/p0111	*bla*_NDM–1_	*bla*_TEM–1B_, *aph(6)-Id*, *aph(3”)-Ib*, *qnrS1*, *sul2*, *tet(A)*, *dfrA14*	OM179752
pST3606-1	1,4,[5],12:i:-	1,09,070	China: Zhuhai	2021/10	IncI1-I(Alpha)	*bla*_NDM–5_	*aadA2*, *dfrA12*, *sul1*	CP094333
pHS36-NDM	Stanley	1,37,952	China	2012/7/25	IncC	*bla*_NDM–1_	*bla*_CMY–6_, *aadA2*, *dfrA12*, *sul1*	KU726616
pRH-1238	Corvallis	1,87,683	Germany	/	IncC	*bla*_NDM–1_	*bla*_CMY–4_, *aph(6)-Id*, *aph(3”)-Ib*, *aadA5*, *aph(3’)-VI*, *aph(3’)-VIa*, *aph(3’)-VIa*, *aac(6’)-Ib3*, *fosA3*, *msr(E)*, *mph(A)*, *mph(E)*, *erm(B)*, *floR*, *sul1*, *sul1*, *sul1*, *sul2*, *tet(A)*, *dfrA17*	KR091911
pSE12-01738-2	Corvallis	1,77,190	Germany	2012	IncC	*bla*_NDM–1_	*bla*_CMY–4_, *aph(6)-Id*, *aph(3”)-Ib*, *aph(3’)-VI*, *aph(3’)-VIa*, *aac(6’)-Ib3*, *fosA3*, *msr(E)*, *mph(E)*, *erm(B)*, *floR*, *sul1*, *sul1*, *sul2*, *tet(A)*	CP027679
AR_0127 plasmid unnamed2	Senftenberg	87,450	/	/	IncM2	*bla*_NDM–1_	*bla*_DHA–1_, *bla*_TEM–1B_, *aac(3)-IId*, *armA*, *msr(E)*, *mph(E)*, *sul1*	CP032193
pAMA003584_NDM-1	Kottbus	42,517	Denmark	2020/11/25	IncN2	*bla*_NDM–1_	/	MZ004973
pS2122_2_NDM-5	1,4,[5],12:i:-	46,161	China: Hangzhou	2022/5/1	IncX3	*bla*_NDM–5_	/	CP110659
p23045-NDM5	1,4,[5],12:i:-	2,66,011	China: Jiaxing	2023/4/23	IncHI2/IncHI2A	*bla*_NDM–5_	*bla*_OXA–10_, *bla*_TEM–1B_, *cmlA1*, *floR*, *lnu(F)*, *sul3*, *tet(A)*, *aadA1*, *aadA2*, *aadA22*, *aph(3”)-Ib*, *aph(3’)-Ia*, *aph(4)-Ia*, *aph(6)-Id*	OR497833
pST_HI2_NDM-1	1,4,[5],12:i:2	3,21,025	China	2020/9/3	IncHI2/IncHI2A	8 copies of *bla*_NDM–1_	*aadA5*, *msr(E)*, *mph(E)*, *sul1*, *tet(B)*, *dfrA17*	CP129631
pC629	Indiana	2,10,106	China	2014/12/5	IncHI2/IncHI2A/IncN	*bla*_NDM–9_	*bla*_OXA–1_, *bla*_TEM–1B_, *bla*_CTX–M–65_, *aph(4)-Ia*, *aadA2*, *aadA5*, *aac(3)-IV*, *rmtB*, *aac(6’)-Ib-cr*, *fosA3*, *mph(A)*, *floR*, *catB3*, *OqxB*, *OqxA*, *ARR-3*, *sul1*, *sul1*, *sul2*, *dfrA12*, *dfrA17*, *bleO*	CP015725
pNDM5_LS002	1,4,[5],12:i:-	1,55,318	China	2022/7/19	IncHI2/IncHI2A	*bla*_NDM–5_	*bla*_OXA–1_, *bleO*, *aac(6’)-Ib-cr*, *floR*, *catB3*, *OqxB*, *OqxA*, *ARR-3*, *dfrA12*	OP290545
pYZPW131	Typhimurium	46,161	/	/	IncX3	*bla*_NDM–5_	/	MK848866
SA17155_ plasmid_ unnamed	1,4,[5],12:i:-	1,99,024	China:Beijing	2022/10/30	IncHI2/IncHI2A	*bla*_NDM–1_	*aph(4)-Ia*, *aac(3)-Iid*, *aadA16*, *aph(3’)-Ia*, *aadA1*, *aadA2b*, *aac(3)-IV*, *aac(6’)-Ib-cr*, *mph(A)*, *cmlA1*, *floR*, *catB3*, *oqxB*, *oqxA*, *ARR-3*, *ARR-3*, *sul1*, *sul1*, *sul1*, *sul2*, *sul3*, tet(A), *dfrA27*, *qacE*, *qacE*	CP123281
p0085-NDM	/	2,39,910	China	/	IncHI2/IncHI2A/IncN	*bla*_NDM–9_	*bla*_OXA–1_, *bla*_CTX–M–65_,*bleO*, *aph(4)-Ia*, *aph(3’)-Ia*, *aadA2*, *aadA5*, *aac(3)-IV*, *aac(6’)-Ib-cr*, *fosA3*, *mph(A)*, *floR*, *catB3*, *OqxB*, *OqxA*, *ARR-3*, *sul1*, *sul1*, *tet(A)*, *dfrA12*, *dfrA17*	MN577015
pNDM5_SH160	1,4,[5],12:i:2	46,161	China: Shanghai	2016/6	IncX3	*bla*_NDM–5_	/	CP053295
1722_ plasmid_ unnamed1	1,4,[5],12:i:-	2,08,610	China: dongyang,zhejiang	2020/11/3	IncHI2/IncHI2A	*bla*_NDM–5_	*bla*_OXA–1_, *aac(6’)-Ib-cr*, *catB3*, *ARR-3*, *sul1*	CP068019
pST2742-1	1,4,[5],12:i:-	2,46,818	China:Zhuhai	2023/07/09	IncHI2/IncHI2A	*bla*_NDM–5_	*aph(4)-Ia*, *aph(6)-Id*, *aph(3’)-Ia*, *aph(3”)-Ib*, *aadA22*, *aadA1*, *aadA2b*, *aac(3)-IV*, *lnu(F)*, *cmlA1*, *floR*, *sul3*, *tet(A)*, *tet(A)*	CP162903
p2024406-NDM5	Stanley	2,77,574	China:Jiaxing	2024/9/3	IncHI2/IncHI2A	*bla*_NDM–5_/*bla*_NDM–5_	*bla*_OXA–10_, *bla*_TEM–1B_, *aadA1*, *aadA1*, *aadA2b*, *aac(3)-IV*, *aph(3’)-Ia*, *aph(4)-Ia*, *aph(6)-Id*, *aph(3”)-Ib*, *cmlA1*, *cmlA1*, *floR*, *qnrS1*, *ARR-2*, *sul3*, *tet(A)*, *tet(A)*, *dfrA14*	PQ844496

Plasmids of different Inc groups, including IncC, IncFII, IncM, IncX3, and IncHI2/IncHI2A, were also found to carry *bla*_NDM_ genes (Table 3), with IncC and IncHI2/IncHI2A being the most common. Plasmids in *S*. Stanley strains, namely pHS36-NDM and p2024406-NDM5 (this study), belong to IncC and IncHI2/IncHI2A, respectively. Multiple copies of *bla*_NDM_ on a single plasmid were found in pSM28_NDM_1 (three copies of *bla*_NDM–1_), pSM30_NDM_1 (seven copies of *bla*_NDM–1_), and pST_HI2_NDM-1 (eight copies of *bla*_NDM–1_). Both pSM28_NDM_1 and pSM30_NDM_1 are IncC-type plasmids, hosted by *S*. Mbandaka. The IncHI2/IncHI2A-type plasmid pST_HI2_NDM-1 was isolated from a 1,4,[5],12:i:2 strain in 2020. Two copies of *bla*_NDM–5_ were found in p2024406-NDM5. Overall, IncHI2/IncHI2A and IncC plasmids carry more resistant genes than IncX3, IncFII, and IncN2 plasmids.

**FIGURE 2:**
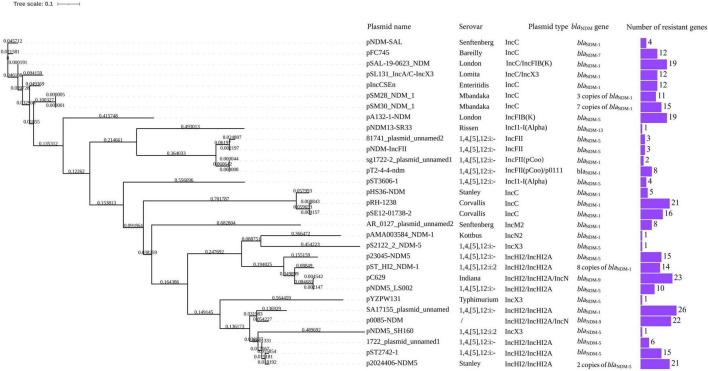
Phylogenetic tree of p23045-NDM5 and p2024406-NDM5 and 29 screened *bla*_NDM_-carrying plasmid of Salmonella isolates from GenBank.

Phylogenetic analysis showed similarity between p23045-NDM5 and pST_HI2_NDM-1 carried by 1,4,[5],12:i:2. Eight copies of *bla*_NDM–1_, in addition to 14 other resistance genes, were found in pST_HI2_NDM-1. The highest homology was between p2024406-NDM5 and pST2742-1, which harbors *bla*_NDM–5_ and was isolated from a 1,4,[5],12:i:- strain. IncHI2/IncHI2A- and IncX3-type plasmids were more similar than IncC- and IncFII-type NDM-positive plasmids in *Salmonella* isolates (Figure 2).

## 4 Discussion

Carbapenems are last-resort antimicrobial agents against infections caused by MDR Gram-negative bacteria. Infection with CRE has become an urgent and continuous threat to public health worldwide (Temkin et al., 2014). Resistance to carbapenems among *Salmonella* isolates is primarily attributed to the presence of mobile genetic elements encoding various classes of β-lactamases. These include carbapenemase, temoneira, NDM, oxacillinase, imipenemase, and Verona integron-encoded metallo-β-lactamase. The carrier isolates with a single copy gene have high minimum inhibitory concentration (MIC) values for all β-lactams (Han et al., 2020, Miao et al., 2018).

In China, the most prevalent carbapenemase gene among *bla*_NDM_-positive isolates is *bla*_NDM–1_, followed by *bla*_NDM–5_ and *bla*_NDM–3_ (Hu et al., 2017). In recent years, *bla*_NDM_ genes have also been identified in rare *Salmonella* serotypes, such as *S*. Kottbus, *S*. Corvallis, and *S*. Lomita (Nielsen et al., 2021, Villa et al., 2015, Li et al., 2020). ST34 *S*. Typhimurium is often characterized by MDR expressed through several resistance genes, including *mcr-1*, *bla*_CTX–M–55_, and *qnrS*. A previous report characterized an ST34 *S*. Typhimurium isolate carrying *bla*_NDM–5_ and the clonal dissemination of *bla*_NDM–1_-positive ST34 *S*. Typhimurium in South China (Deng et al., 2024). Here, we have described the first identification of *Salmonella* isolates carrying *bla*_NDM_ in Jiaxing City. Our isolation of two unrelated clinical *Salmonella* isolates of different serovars, both carrying *bla*_NDM–5_, indicates that the major NDM type in Jiaxing is NDM-5.

Horizontal transmission mediated by various Inc groups of plasmids constitute the major route for the ongoing spread of carbapenem resistance, and include IncC, IncC/IncFIB(K), IncC/IncX3, IncFIB(K), IncI1-I(Alpha), IncFII, IncFII(pCoo), IncFII(pCoo)/p0111, IncM2, IncN2, IncX3, IncHI2/IncHI2A, and IncHI2/IncHI2A/IncN. All of these were isolated from different serotypes across various countries from 2012 to 2024, highlighting the global burden of *bla*_NDM_-positive plasmid in *S. enterica*. Most IncHI2 plasmids found in ST34 *S*. Typhimurium strains shared a similar backbone, with the capture of *bla*_NDM–1_ through an IncHI2/ST3 plasmid (Deng et al., 2024). Although IncX3 has been deemed the primary vehicle for *bla*_NDM_ transmission worldwide in *Enterobacteriaceae* (Guo et al., 2019), IncHI2/ST3 plasmids have replaced IncX3 plasmids as the primary plasmid vector for *bla*_NDM–5_ transmission on some farms (He et al., 2023).

ST3-IncHI2 plasmids exhibit high sequence conservation in backbones, but possess highly genetic plasticity in accessory regions, allowing for the acquisition of numerous antibiotic resistance genes through mobile elements (Fang et al., 2018). Many mobile elements have played crucial roles in the dissemination of *bla*_NDM_, including IS*26*, IS*Aba125*, IS*5*, IS*CR1*, Tn*3*, Tn*125*, and Tn*3000* (Feng et al., 2018, Zhao et al., 2021, Li et al., 2021). A novel IS*26*-flanked composite transposon (Tn*7540*) in the chromosome of an *S*. Indiana isolate was found to carry *bla*_NDM–9_ and *fosA3* (Sun et al., 2023). An IS*15DIV*-flanked composite transposon also contributed to the dissemination of *bla*_NDM–5_ in *S*. Typhimurium (Zhao et al., 2025). NDM-positive isolates consistently carry either a complete or fragmented IS*Aba125*, providing a promoter region for *bla*_NDM_ and playing a critical role in the horizontal transmission of *bla*_NDM–5_ and other resistance determinants (Zhao et al., 2025). Our comparative plasmid analysis showed that the deletion of IS*Aba125* may have been occurred late in the evolution of p23045-NDM5. Up to eight tandem copies of an IS*CR1* unit (IS*CR1*-*dsbD*-*trpF*-*ble*-*bla*_NDM–1_-ΔIS*Aba125*) were found on an HI2 plasmid in *S*. Typhimurium (Song et al., 2023). Although plasmid-borne *bla*_NDM–5_ is usually found as a single copy, we previously identified two non-tandem copies of *bla*_NDM–5_ on a 144,225-bp IncF plasmid from a carbapenem-resistant clinical isolate of *E. coli* (Feng et al., 2018). The coexistence of two *bla*_NDM–5_ genes was attributed to duplication of an IS*26*-bracketed region containing IS*CR1*. In the present study, the two *bla*_NDM–5_ regions within one IncHI2/IncHI2A plasmid carried by *S*. Stanley may have resulted from the duplication of a unit comprising IS*26*-*umuD*-*umuC*-△ IS*Kox3*-△ IS*3000*-△ IS*Aba125*-IS*5*-*bla*_NDM–5_-*ble*_MBL_-*trpF*-*dsbC*-IS*26* that was subsequently interrupted by IS*1* upstream of △ IS*3000.* No IS*CR1* sequences were found in p23045-NDM5 or p2024406-NDM5.

## 5 Conclusion

In conclusion, we described two MDR *Salmonella* strains carrying *bla*_NDM–5_ that were isolated in Jiaxing City, China, specifically 2023JX045 (4,[5],12:i:-) and 2024-406 (*S*. Stanley). Each strain was shown to harbor a *bla*_NDM–5_-positive IncHI2/IncHI2A plasmid (p23045-NDM5 in 2023JX045 and p2024406-NDM5 in 2024-406), exhibiting signs of multiple evolutionary events that contributed to the diversity of the *bla*_NDM–5_-region. IS*26*-flanked composite transposons appeared to play an important role in the formation of this region. The complex diversity of the *bla*_NDM–5_ region is one explanation for the common development of MDR host strains. To the best of our knowledge, this is the first report of a *bla*_NDM_ gene carried by *Salmonella*, a major foodborne pathogen, in this region of China. Importantly, this is also the first report of a single IncHI2/IncHI2A plasmid carrying two copies of *bla*_NDM–5_ in an *S*. Stanley host. The identification of CRSE isolates harboring *bla*_NDM_ and the expanding diversity of *bla*_NDM–5_-positive plasmids indicate the potential for widespread dissemination.

This study has several limitations. Since only two isolates were analyzed in this study, the transmission and evolution mechanism of NDM in *Salmonella* has not been fully explained. The sources of infection of the two cases were also not successfully identified. Therefore, we recommend heightened vigilance and international cooperation to mitigate the public health impact of these pathogens.

## Data Availability

The datasets presented in this study can be found in online repositories. The names of the repository/repositories and accession number(s) can be found in the article/supplementary material.

## Footnotes

1
http://denglab.info/SeqSero2

2
https://www.pubmlst.org

3
https://cge.food.dtu.dk/services/ResFinder/

4
https://cge.food.dtu.dk/services/PlasmidFinder/

5
https://www.is.biotoul.fr

6
https://mjsull.github.io/Easyfig/

7
https://itol.embl.de/
